# Obesity, adiposopathy, and quantitative imaging biomarkers

**DOI:** 10.1590/0100-3984.2017.50.3e2

**Published:** 2017

**Authors:** Fernando Ide Yamauchi, Adham do Amaral e Castro

**Affiliations:** 1 MD, Attending Radiologist at Instituto de Radiologia do Hospital das Clínicas da Faculdade de Medicina da Universidade de São Paulo (InRad/HC-FMUSP), Attending Radiologist in the Department of Radiology, Hospital Israelita Albert Einstein, São Paulo, SP, Brazil. (E-mail: fernando.yamauchi@hc.fm.usp.br).; 2 PhD, MD, Attending Radiologist in the Department of Radiology, Hospital Israelita Albert Einstein, Attending Radiologist in the Department of Diagnostic Imaging , Escola Paulista de Medicina da Universidade Federal de São Paulo (EPM-Unifesp), São Paulo, SP, Brazil.

Obesity is a metabolic disease with increasing incidence at a global level. The prevalence of obesity doubled between 1980 and 2014, now corresponding to more than half a billion obese people worldwide^(1)^. The World Health Organization estimates that more than a third of adults over 18 years of age are now overweight.

Obesity plays an important role in the development of several diseases, such as atherosclerosis, diabetes, musculoskeletal conditions (e.g., osteoarthritis, tendinopathy, and carpal tunnel syndrome), and chronic pain^(2-5)^. Another important association is the increased risk of cancer^(6,7)^. The development of these conditions is likely related to increased production of pro-inflammatory adipokines (e.g., interleukin 6 and tumor necrosis factor alpha) and decreased production of (or decreased tissue sensitivity to) anti-inflammatory adipokines (e.g., adiponectin). The final result is that those individuals are in an inflammatory state and show increased levels of acute phase reagents such as C-reactive protein^(8)^.

In the field of radiology, there is a trend toward more quantitative science that could increase the value of quantitative imaging biomarkers and reduce variability across devices, patients, and time. A quantitative imaging biomarker can be defined as “an objective characteristic derived from an *in vivo* image measured on a ratio or interval scale as indicators of normal biological processes, pathogenic processes, or a response to a therapeutic intervention”^(9,10)^. It is extremely important that measurements can be reproduced by different observers on different equipment. In this context, the Radiological Society of North America has organized a Quantitative Imaging Biomarker Alliance.

There is great interest in quantitative measurements of adipose tissue, to serve as imaging biomarkers. Total body adipose tissue can be better understood and quantified through sectional imaging methods such as computed tomography and magnetic resonance imaging. It can be divided into two main categories: subcutaneous and internal. Internal fat can be further divided into two components: visceral and nonvisceral. The visceral component includes the adipose tissue distributed in three body cavities: thoracic, intra-abdominal, and pelvic. The nonvisceral component includes intermuscular and paravertebral adipose tissue^(11)^.

Recent studies have demonstrated that deposition of visceral fat is an important imaging biomarker of metabolic disease^(12,13)^, linked to the concept of adiposopathy, also known as sick fat syndrome. Adiposopathy can be defined as “a pathologic adipose tissue anatomic/functional disturbances promoted by positive caloric balance in genetically and environmentally susceptible individuals which results in adverse endocrine and immune responses that both directly and indirectly contribute to metabolic disease and increased cardiovascular disease risk”^(14)^.

In an article published in this issue of **Radiologia Brasileira**, Mauad et al. proposed using ultrasound and computed tomography to quantify abdominal fat and found correlations with body mass index, serum cholesterol, and abdominal circumference^(15)^. Although their study has certain limitations, the authors suggest that ultrasound might be used as an alternative method for abdominal fat quantification, with advantages including its wide availability, its lower cost, and the fact that it does not involve the use of ionizing radiation. It is important to notice that, in order to be considered suitable for quantitative imaging biomarkers, ultrasound measurements should be further correlated with cardiovascular events.
